# Selective Modulation of MicroRNA Expression with Protein Ingestion Following Concurrent Resistance and Endurance Exercise in Human Skeletal Muscle

**DOI:** 10.3389/fphys.2016.00087

**Published:** 2016-03-07

**Authors:** Donny M. Camera, Jun N. Ong, Vernon G. Coffey, John A. Hawley

**Affiliations:** ^1^Mary MacKillop Institute for Health Research, Centre for Exercise and Nutrition, Australian Catholic UniversityMelbourne, VIC, Australia; ^2^Exercise and Nutrition Research Group, School of Medical Sciences, Royal Melbourne Institute of TechnologyMelbourne, VIC, Australia; ^3^Bond Institute of Health and Sport and Faculty of Health Sciences and Medicine, Bond UniversityGold Coast, QLD, Australia; ^4^Research Institute for Sport and Exercise Sciences, Liverpool John Moores UniversityLiverpool, UK

**Keywords:** anabolic, metabolic, adaptation, molecular, resistance exercise, endurance exercise

## Abstract

We examined changes in the expression of 13 selected skeletal muscle microRNAs (miRNAs) implicated in exercise adaptation responses following a single bout of concurrent exercise. In a randomized cross-over design, seven healthy males undertook a single trial consisting of resistance exercise (8 × 5 leg extension, 80% 1 Repetition Maximum) followed by cycling (30 min at ~70% VO_2peak_) with either post-exercise protein (PRO: 25 g whey protein) or placebo (PLA) ingestion. Muscle biopsies *(vastus lateralis)* were obtained at rest and 4 h post-exercise. Detection of miRNA via quantitative Polymerase Chain Reaction (qPCR) revealed post-exercise increases in miR-23a-3p (~90%), miR-23b-3p (~39%), miR-133b (~80%), miR-181-5p (~50%), and miR-378-5p (~41%) at 4 h post-exercise with PRO that also resulted in higher abundance compared to PLA (*P* < 0.05). There was a post-exercise decrease in miR-494-3p abundance in PLA only (~88%, *P* < 0.05). There were no changes in the total abundance of target proteins post-exercise or between conditions. Protein ingestion following concurrent exercise can modulate the expression of miRNAs implicated in exercise adaptations compared to placebo. The selective modulation of miRNAs with target proteins that may prioritize myogenic compared to oxidative/metabolic adaptive responses indicate that miRNAs can play a regulatory role in the molecular machinery enhancing muscle protein synthesis responses with protein ingestion following concurrent exercise.

## Introduction

Individuals participating in fitness programs undertake concurrent resistance and endurance exercise as part of their training. Given the divergent phenotypes generated by single mode endurance and resistance training, there is inherent potential for “interference” in exercise adaptation responses when these exercise modes are performed concurrently (Wilson et al., 2012). We recently reported increased rates of myofibrillar protein synthesis (MPS) with protein compared to placebo ingestion following an acute bout of concurrent resistance and endurance exercise, showing that increased protein availability can further promote the anabolic response when combining divergent exercise modes (Camera et al., 2015).

The regulation of exercise-induced responses to concurrent exercise has primarily focused on changes in markers of gene transcription and translation (Coffey et al., 2009a; Camera et al., 2015). However, emerging evidence suggests a role for microRNAs (miRNAs) in the control of exercise adaptation responses through alterations in mRNA expression (Kirby and McCarthy, 2013). miRNAs, small (~20–30 nucleotides) non-coding ribonucleic acids (RNAs), promote messenger RNA (mRNA) degradation and suppress or inhibit protein translation (He and Hannon, 2004). As a result, the modulation of specific miRNA(s) has the capacity to mediate changes in expression levels of particular mRNAs which may contribute to the specificity of adaptation responses following exercise (Kirby and McCarthy, 2013). Moreover, individual miRNAs can target hundreds of mRNAs while individual mRNAs can also be subject to regulation by multiple miRNAs (Kirby and McCarthy, 2013).

Myomir's are a large group of skeletal muscle-enriched miRNAs implicated in the response to exercise. Previous studies show miRNA responses regulating “myogenic markers” have altered expression profiles between young and old individuals (Drummond et al., 2008; Rivas et al., 2014; Zacharewicz et al., 2014) as well as between “high” and “low” responders following resistance training (Davidsen et al., 2011). Similarly, differences in specific miRNAs regulating mitochondrial biogenesis and substrate metabolism have been reported following acute (Nielsen et al., 2010; Russell et al., 2013) and chronic (Nielsen et al., 2010; Russell et al., 2013) aerobic exercise. No study has investigated the miRNA response when resistance and endurance exercise are combined.

Limited evidence also exists on the effect of increased protein availability on human skeletal muscle miRNA expression with only one study reporting increased expression of selected myomir's following amino acid ingestion (Drummond et al., 2009). The effect of protein ingestion on miRNA expression following exercise has not been determined. Accordingly, the aims of this study were to (a) characterize the expression of select miRNAs previously shown to be modulated by either resistance or endurance exercise following a bout of concurrent resistance and endurance exercise; and (b) determine the effects of protein compared to placebo ingestion on miRNA expression abundance and selected target proteins following concurrent exercise. Given the evidence for protein ingestion to modulate MPS responses following concurrent exercise (Camera et al., 2015), we hypothesized this would mediate a miRNA expression profile prioritizing anabolic rather than oxidative/metabolic adaptations when endurance exercise is undertaken after a prior bout of resistance exercise.

## Materials and methods

### Subjects

Seven physically fit male subjects (19.3 ± 1.4 year, body mass [BM] 77.6 ± 16.8 kg, peak oxygen uptake (VO_2peak_) 47.5 ± 4.0 mL/kg/min, leg extension one repetition maximum (1 RM) 127.9 ± 14.7 kg; values are mean ± Standard Deviation [SD]) volunteered to participate in this study as previously described (Camera et al., 2015). Upon explanation of the experimental procedures and possible risks associated with the study, subjects provided written informed consent before participation. We chose to study subjects with a history of concurrent resistance and aerobic-based training (~3/week; > 1 year) to avoid any “novel” responses in skeletal muscle following a bout of concurrent training. The study was approved by the Human Research Ethics Committee of RMIT University and with the 1964 Helsinki declaration and its later amendments or comparable ethical standards.

### Study design

The study employed a randomized counter-balanced, double-blind, cross-over design in which each subject completed two bouts of concurrent resistance exercise and cycling with either post-exercise placebo (PLA) or protein (PRO) ingestion separated by a 3 week recovery period, during which time subjects maintained their habitual physical activity pattern.

### Preliminary testing

All subjects underwent preliminary strength and aerobic capacity testing 2 weeks before an experimental trial as described previously (Camera et al., 2015). Briefly, maximal quadriceps strength was determined using a series of single repetitions on a plate-loaded leg extension machine until the maximum load lifted was established (1RM). VO_2peak_ was determined via an incremental test to volitional fatigue on a Lode cycle ergometer (Groningen, The Netherlands) where subjects commenced cycling at a workload equivalent to 2W/kg for 150 s with subsequent 25 W increments in workload every 150 s (Camera et al., 2015).

### Diet and exercise control

Subjects were instructed to refrain from all exercise training and vigorous physical activity, and alcohol and caffeine consumption for a minimum of 48 h prior to an experimental trial. Standardized pre-packed meals comprising 3 g carbohydrate/kg BM, 0.5 g protein/kg BM and 0.3 g fat/kg BM were provided to the subjects to be consumed as the final evening meal before reporting for the trial.

### Experimental trials

Subjects reported to the laboratory after a ~ 10 h overnight fast. Under local anesthesia (2–3 mL of 1% Xylocaine) a resting muscle biopsy was obtained from the *vastus lateralis* using a 5 mm Bergstrom needle modified with suction. After a 10 min rest subjects completed a standardized warm up (2 sets of 5 repetitions at ~50 and ~60% 1 RM, respectively) on a leg extension machine. Subjects then completed the main resistance exercise protocol which consisted of eight sets of five repetitions leg extension at ~80% 1RM. Each set was separated by a 3 min recovery period during which time the subjects remained seated on the leg extension machine. Subjects then rested for 15 min before commencing 30 min of continuous cycling at a power output that elicited 70% of individual VO_2peak_. Immediately following the cessation of exercise, subjects ingested 500 mL of either a placebo (PLA: water, artificial sweetener) or protein beverage (PRO: 25 g whey protein). Subjects then rested for 240 min after which time an additional muscle biopsy was obtained. Resistance exercise was performed prior to endurance exercise as it has been previously reported that the acute anabolic responses are attenuated when endurance exercise precedes resistance exercise (Coffey et al., 2009a). Our choice of a 15 min recovery between resistance and endurance exercise sessions was based on anecdotal reports from coaches of elite athletes regarding selected training regimens in a number of sports and has been used previously by our laboratory (Coffey et al., 2009a,b). Given the inherent difficulties in matching the total work undertaken between different exercise modes and when comparing with a concurrent exercise bout we chose to characterize the acute concurrent training response in isolation.

### RNA extraction and quantification

Skeletal muscle tissue RNA extractions were performed using a TRIzol-based kit according to the manufacturer's protocol (Invitrogen, Melbourne, Australia). Briefly, ~20 mg of skeletal muscle was homogenized in TRIzol and chloroform added to form an aqueous RNA phase. This RNA phase was then precipitated by mixing with isopropanol alcohol and the resulting pellet was washed and re-suspended in RNase-free water. Extracted RNA was quantified using a NanoDrop 1000 spectrophotometer (Thermo Fisher, MA, USA) by measuring absorbance at 260 nm and 280 nm with a 260/280 ratio of 1.7–1.9 recorded for all samples.

### Reverse transcription (RT) and real-time PCR

For miRNA analyses, 250 ng RNA was reverse transcribed using a miScript II RT Kit (Qiagen, Australia) in a BioRad thermal cycler (BioRad, Gladesville, Australia) according to the manufacturer's protocol. All samples then underwent a quality control (QC) using a 96-well miScript miRNA QC PCR Array (Cat No. MIHS-989Z, Qiagen, Australia) in a 96-well RT cycler CFX96 (BioRad, Gladesville, Australia). All samples passed QC with threshold cycles (Ct) for the positive PCR Control between 17 and 21 and duplicates within 0.5 Ct. Quantification of miRNA (in duplicate) was performed on a customized 96-well miScript miRNA PCR Array from Qiagen (Custom Cat Number: CMIHS02243) under the same cycling conditions as in the QC. The array contained 13 common miRNAs previously shown in the literature to be regulated following resistance or endurance exercise, and amino acid ingestion in human skeletal muscle including hsa-miR-1, hsa-miR-9-3p, hsa-miR-16-5p, hsa-miR-23a-3p, hsa-miR-23b-3p, hsa-miR-31-5p, hsa-miR-133a-3p, hsa-miR-133b, hsa-miR-181a-5p, hsa-miR-378a-5p, has-miR-451a, hsa-miR-486-5p, and hsa-miR-494-3p. We undertook this “targeted” expression approach to reduce any potential for type 2 errors (false positives) that may occur if analysing a broader, non-specific spectrum of miRNAs. RNU48 was used a housekeeping control based on limited previous studies (Davidsen et al., 2011; Russell et al., 2013) within the array and there were no changes in expression post-exercise (*P* > 0.05). The 2^ΔΔCT^ method of relative quantification was used to calculate the relative amounts of miRNAs (Livak and Schmittgen, 2001).

### Selection of predicted and validated miRNA targets

The TargetScanHuman™ (Release 7.0:August 2015) bioinformatics algorithm (Agarwal et al., 2015) was used for miRNA target prediction to identify mRNA/protein targets for miRNAs altered post-exercise or between PLA and PRO groups based on statistical significance (*P* < 0.05). The selection of protein targets for analysis were limited to those previously implicated in adaptation responses to either resistance or endurance exercise. Total protein levels of these targets were verified with Western Blot analysis (described subsequently).

### Western blotting

Approximately 30 mg of tissue was homogenized in ice-cold buffer containing 50 mM of Tris–HCl, pH 7.5, 1 mM of EDTA, 1 mM of EGTA, 10% glycerol, 1% Triton X-100, 50 mM of NaF, 5 mM of sodium pyrophosphate, 1 mM of DTT, 10 μg/mL of trypsin inhibitor, 2 μg/mL of aprotinin, 1 mM of benzamidine, and 1 mM PMSF using a motorized pellet pestle (Sigma-Aldrich, St. Louis, MO) with 5 s pulses. The lysate was kept on ice at all times and was then centrifuged at 12,000 g for 20 min at 4°C (Camera et al., 2015). Working lysates were re-suspended in Laemmli sample buffer with 40 μg of protein loaded onto 4–20% Mini-PROTEAN TGX Stain-Free™Gels. Post electrophoresis, gels were activated according to the manufacturer's details (Bio-Rad, Gladesvilles, Australia) and then transferred to polyvinylidine fluoride (PVDF) membranes. After transfer, a Stain-Free image of the PVDF membranes for total protein normalization was obtained before membranes were rinsed briefly in distilled water and blocked with 5% non-fat milk/ TBS, and incubated with primary antibody (1:1000) overnight at 4°C. Membranes were incubated with secondary antibody (1:2000), and proteins were detected via enhanced chemiluminescence (Pierce, MA, USA) and quantified by densitometry (ChemiDoc™ XRS+ System; Bio Rad, USA). All sample time points for each subject in both PLA and PRO conditions were run on the same gel. Polyclonal Histone Deacetylase 4 (HDAC4) (#2072), Nuclear Respiratory Factor 1 (NRF1) (#12381), Sirtuin 1 (SIRT1) (#2310), and monoclonal Glycogen Synthase Kinase β (GSK3β) (#9315), Forkhead box O1 (FOXO1) (#2880) and FOXO3a (#2497) were purchased from Cell Signalling Technology (Danvers, USA). Volume density of each target protein band was normalized to the total protein loaded into each lane using stain-free technology (Gurtler et al., 2013) with data expressed in arbitrary units.

### Statistical analysis

miRNA and cell signaling data were analyzed by two-way ANOVA (two factor: time × treatment) with repeated measures with Student-Newman-Keuls *post-hoc* analysis when *P* < 0.05 (SigmaStat for windows Version 3.11). Linear regression (Graph Pad Prism 6.0) was performed to define any correlations between post-exercise changes in miRNAs and their predicted protein targets. All data are expressed as mean ± SD.

## Results

### miRNA expression

**Figure 1:**
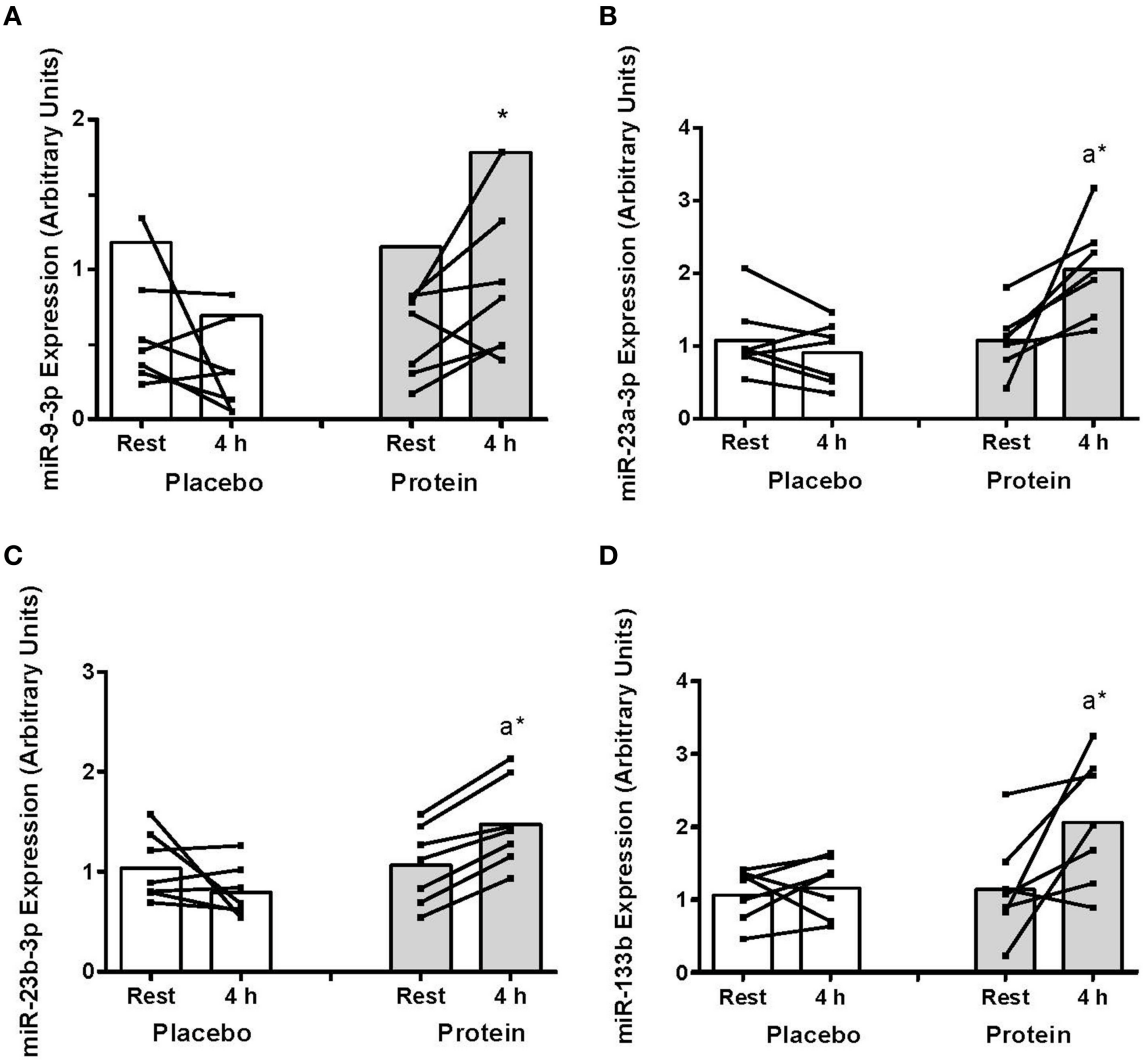
**(A) mir-9-3p, (B) miR-23a-3p, (C) miR-23b-3p, and (D) miR-133b abundance at rest and at 4 h post-exercise recovery following a concurrent exercise session of resistance (8 sets of 5 leg extension at 80% 1-RM) and endurance (30 min cycling at 70% VO_2peak_) exercise and ingestion of either 500-mL PLA or PRO beverage immediately after exercise**. Values are arbitrary units expressed relative to RNU48 and presented as individual data with group mean (*n* = 7). Significantly different (*P* < 0.05) vs. (a) rest and (^*^) between treatments (PLA vs. PRO).

There was a decrease in miR-9 expression with PLA post-exercise that resulted in higher miR-9 with PRO compared to PLA at 4 h (~150%, *P* < 0.05; Figure 1A). There were increases in miR-23a and miR-23b abundance above rest at 4 h only in PRO (~38–90%; *P* < 0.05) that resulted in higher expression at 4 h with PRO compared to PLA (85–127%, *P* < 0.05; Figures 1B,C). The expression of miR-133b also increased post-exercise only with PRO (~75%, *P* < 0.05, Figure 1D).

**Figure 2:**
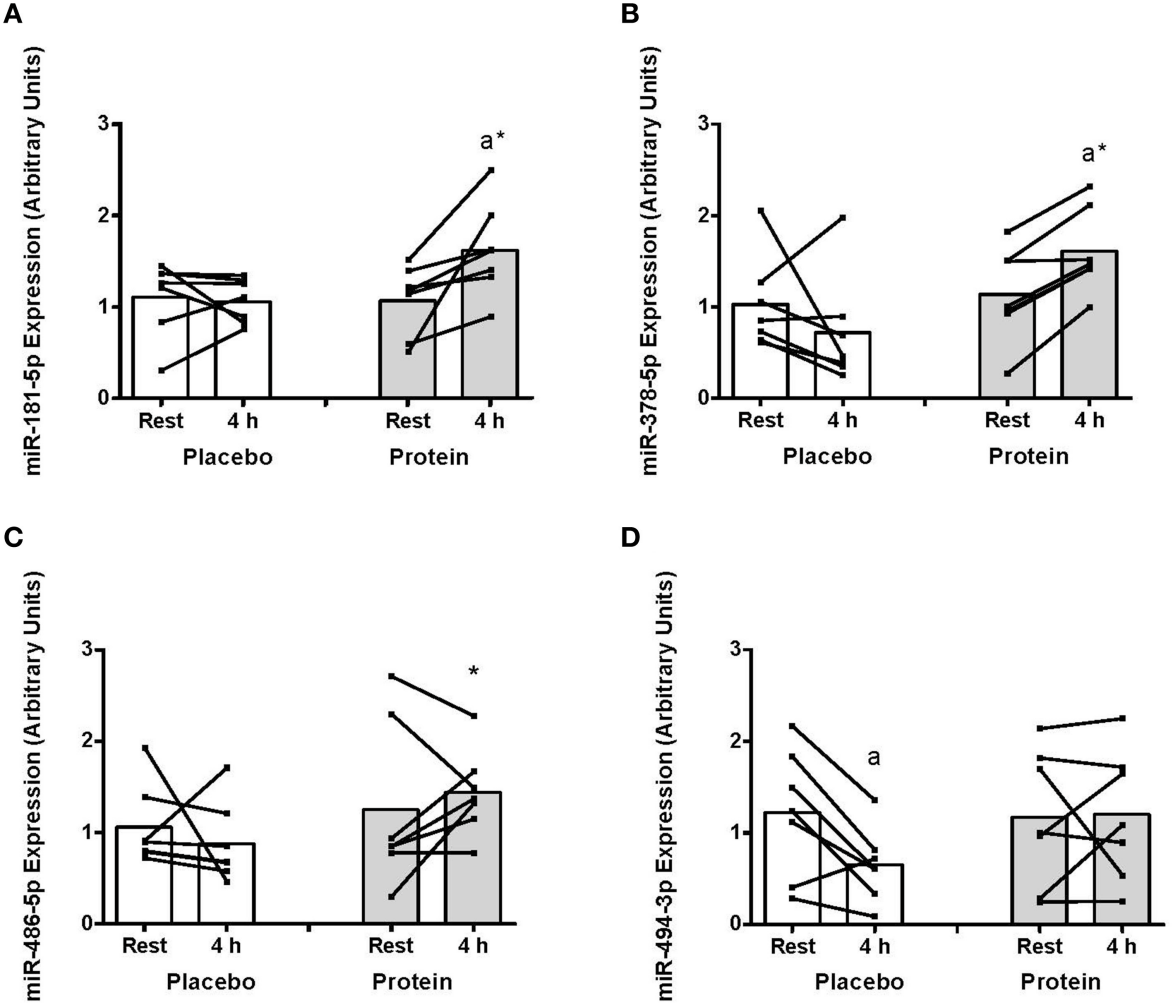
**(A) mir-181-5p, (B) miR-378-5p, (C) miR-486-5p, and (D) miR-494-3p abundance at rest and at 4 h post-exercise recovery following a concurrent exercise session of resistance (8 sets of 5 leg extension at 80% 1-RM) and endurance (30 min cycling at 70% VO_2peak_) exercise and ingestion of either 500-mL PLA or PRO beverage immediately after exercise**. Values are arbitrary units expressed relative to RNU48 and presented as individual data with group mean (*n* = 7). Significantly different (*P* < 0.05) vs. (a) rest and (^*^) between treatments (PLA vs. PRO).

The expression of miR-181 increased at 4 h post-exercise with PRO (~80%, *P* < 0.05) that resulted in higher miR-181 with PRO compared to PLA at 4 h (~76%, *P* < 0.05; Figure 2A). There was an increase in miR-378 abundance in PRO from rest to 4 h (~40%, *P* < 0.05) and higher miR-378 with PRO above PLA at 4 h (~124%, *P* < 0.05, Figure 2B). There were no post-exercise changes in miR-486 with PLA or PRO although miR-486 expression was higher at 4 h with PRO compared to PLA (~63%, *P* < 0.05, Figure 2C). miR-494 decreased at 4 h post-exercise only with PLA (~87%, *P* < 0.05; Figure 2D).

There were no changes post-exercise or between groups for miR-1, -16, -31, -133a, and -451a (Table 1).

**Table 1:** **Expression of unchanged miRNAs measured at rest and 4 h post-exercise in PLA and PRO conditions (Arbitrary Units; mean ± SD)**.

	**PLA**		**PRO**	
**miRNA**	**Rest**	**4 h**	**Rest**	**4 h**
miR-1	1.21 ± 0.72	1.13 ± 0.81	1.31 ± 0.95	1.34 ± 1.01
miR-16	1.04 ± 0.39	1.21 ± 0.93	1.14 ± 0.58	1.30 ± 0.36
miR-31	1.04 ± 0.29	1.24 ± 0.88	1.06 ± 0.39	1.27 ± 0.91
miR-133a	1.11 ± 0.74	0.91 ± 0.71	1.11 ± 0.57	0.97 ± 0.67
miR-451a	1.14 ± 0.57	1.61 ± 0.96	1.15 ± 0.91	1.59 ± 0.94

### Protein expression

**Figure 3:**
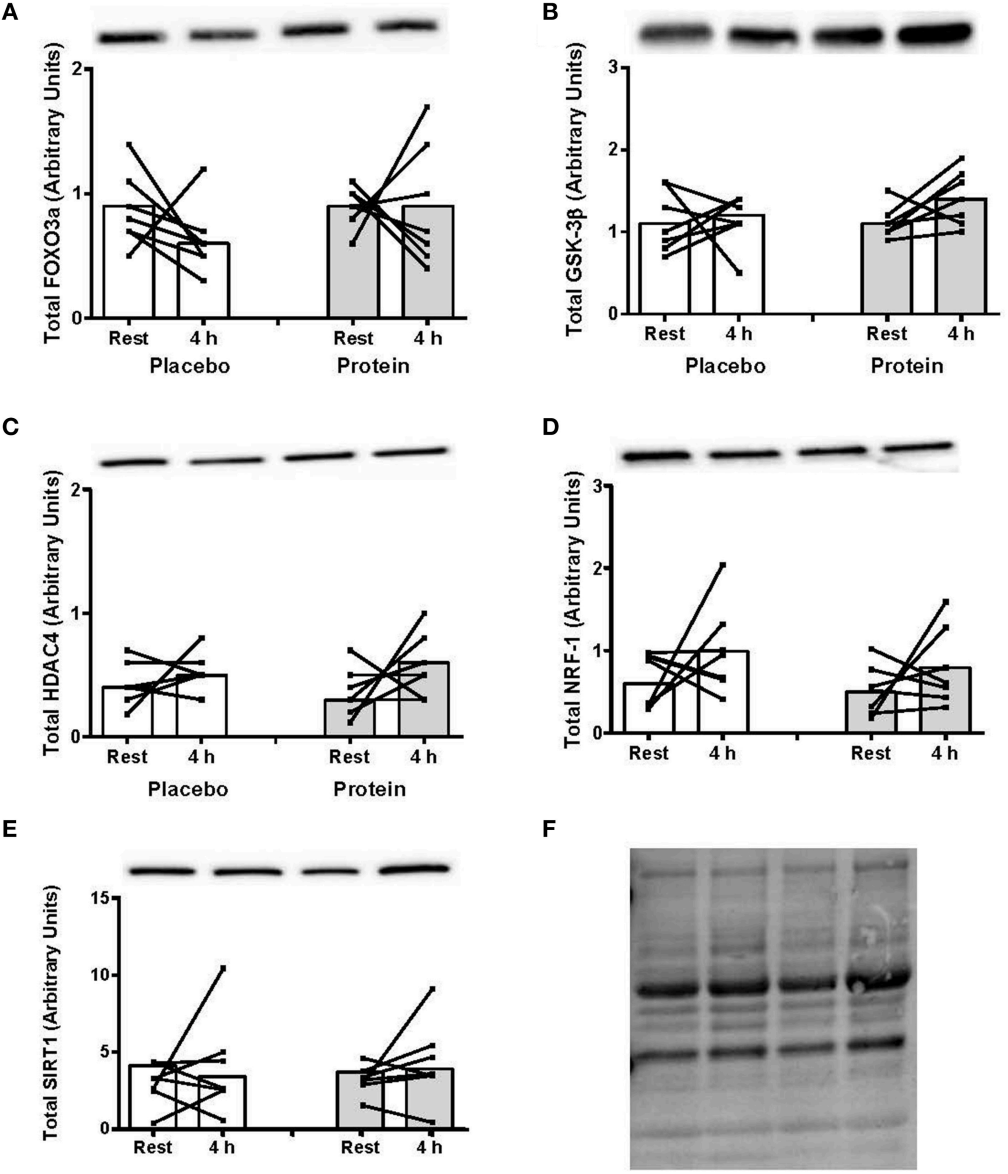
**(A) Foxo3a, (B) GSK-3β, (C) HDAC4, (D) NRF-1, and (E) SIRT1 total protein content at rest and at 4 h post-exercise recovery following a concurrent exercise session of resistance (8 sets of 5 leg extension at 80% 1-RM) and endurance (30 min cycling at 70% VO_2peak_) exercise and ingestion of either 500-mL PLA or PRO beverage immediately after exercise**. Values are arbitrary units expressed relative to RNU48 and presented as individual data with group mean (*n* = 7). **(F)** Stain-free image of total protein loading.

The TargetScanHuman™ (Release 7.0:August 2015) bioinformatics algorithm (Agarwal et al., 2015) was used to identify protein targets for the eight miRNAs significantly altered post-exercise or between PLA and PRO conditions. The predicted targets selected for protein analysis were those that have been shown to be regulated by resistance or endurance exercise, as well as protein ingestion, and those which are implicated in exercise-induced anabolic and metabolic responses including FOXO3 (Sanchez et al., 2014), GSK-3β (Camera et al., 2010), HDAC4 (McGee and Hargreaves, 2011), SIRT1 (Philp and Schenk, 2013), and NRF-1 (Scarpulla et al., 2012). There were no post-exercise changes or differences between PLA and PRO conditions in total protein levels for any of these signaling targets (Figure 3).

### Correlations

We investigated whether there were any correlations between the expression of miRNAs and protein abundance of their predicted target(s). No correlations were observed between any of the altered miRNAs and the expression of their predicted protein(s) (*P* > 0.05; *R*^2^: 0.1–0.4).

## Discussion

miRNAs have recently emerged as potential mediators of skeletal muscle adaptation responses to exercise through their regulation of mRNA expression (Kirby and McCarthy, 2013). This is the first study to show protein ingestion after a concurrent exercise bout promotes miRNA expression in skeletal muscle compared to a post-exercise placebo beverage. This modulation of miRNAs with protein ingestion provides new information on the molecular mechanism that may promote increased MPS responses following concurrent resistance and endurance exercise with protein ingestion.

Studies of concurrent training protocols generally report that endurance exercise attenuates hypertrophy/strength but resistance exercise has little or no negative impact on endurance adaptation (Hickson, 1980; Wilson et al., 2012). The mechanism/s responsible for this anabolic “interference” has yet to be clearly defined. We identified five miRNAs in the current study that increased from rest to post-exercise only with protein ingestion while two other miRNAs were differentially expressed compared to placebo at the 4 h recovery time point. These miRNAs have previously been implicated in exercise adaptation responses to resistance or endurance exercise due to their putative regulation of anabolic and/ or metabolic signaling protein targets. Specifically, miR-23a and 23b were higher post-exercise with protein ingestion which supports previous reports of increased skeletal muscle miR-23a abundance following amino acid ingestion (Drummond et al., 2009). Bioinformatics analysis identified GSK-3β as a target of both miR-23 miRNAs. Phosphorylation of GSK-3β activates eIF2Bε which catalyzes the exchange of GDP for GTP to promote translation initiation via eIF2 (Jefferson et al., 1999). The increased abundance of miR-23a/b may therefore indicate a regulatory mechanism by which protein ingestion can promote increases in translation initiation, and in turn MPS, through reducing GSK-3β's inhibition of eIF2Bε.

We also found greater miR-133b and miR-181 abundance with post-exercise protein ingestion. The histone deacetylase SIRT1 is a predicted target of these miRNAs and SIRT1 is implicated in the regulation of PGC-1α and subsequent transcription of genes associated with mitochondrial metabolism in skeletal muscle (White and Schenk, 2012). While SIRT1 expression increases following endurance exercise (Edgett et al., 2013), we observed no changes in either condition with concurrent exercise nor was there any association between each miRNA and SIRT1. A primary regulator of exercise-induced SIRT1 expression is NAD^+^ availability through AMPK (White and Schenk, 2012) and it seems plausible that AMPK, rather than miR-133b or miR-181, may be the primary regulator of post-exercise changes in SIRT1 expression. Moreover, the miRNA regulation of mRNA targets is dependent on factors such as rates of transcription and translation of selected protein targets and rate of mRNA degradation (Hausser et al., 2013). Given the limited characterization of the regulation and time-course of miRNA expression in human skeletal muscle, it is possible our sampling time-points do not provide the necessary temporal resolution for determining association between miRNAs and protein expression.

The miR-378 was exclusively upregulated with post-exercise protein ingestion and was higher compared to placebo. Gene ontology analysis has revealed miR-378 to target the mTOR signaling pathway while changes in miR-378 expression correlated with increases in lean body mass following 12 weeks resistance training (Davidsen et al., 2011). DEP domain containing MTOR-interacting protein (DEPTOR) is an endogenous inhibitor of mTORC1 and predicted target of miR-378. In light of previous findings (Camera et al., 2015), the increase in miR-378 expression with protein provides a potential mechanism by which protein ingestion after concurrent exercise enhances mTORC1 signaling and rates of MPS. The transcription factor FOXO3 is also a predicted target of miR-378. FOXO3 can regulate muscle protein breakdown through ubiquitin–proteasome activity, and particularly MURF1 and Atrogin transcription (Sanchez et al., 2014). We previously showed protein ingestion after concurrent exercise reduced the mRNA expression of these ubiquitin ligases compared to placebo (Camera et al., 2010). While there were no changes in FOXO3 abundance in the current study, it is possible the miR-378 regulation of FOXO3 may have mediated changes in MURF1 and Atrogin transcription at an earlier time point.

The miR-494, previously implicated in mitochondrial biogenesis responses (Yamamoto et al., 2012), was the only miRNA to decrease post-exercise, an effect that was only evident in the placebo group. Yamamoto and colleagues recently reported miR-494 negatively regulates the expression of the mitochondrial transcription factor A and mitochondrial biogenesis in C_2_C_12_ myoblasts (Yamamoto et al., 2012). Moreover, decreased miR-494 expression has also been associated with increased PGC-1α mRNA following acute swimming exercise in mice (Yamamoto et al., 2012). While a direct interaction between these protein targets has yet to be validated in human skeletal muscle, increases in PGC-1α mRNA and protein expression are commonly observed after endurance exercise in humans (Perry et al., 2010; Little et al., 2011) and we have previously shown PGC-1α mRNA to increase following concurrent exercise (Coffey et al., 2009b; Camera et al., 2015). The exercise-induced down regulation of miR-494 without protein may be an important regulatory step in the molecular machinery promoting mitochondrial-based adaptation following divergent contractile activity. The miR-486 may similarly mediate mitochondrial based adaptations to exercise given its predicted regulation of the transcription factor NRF-1. Notwithstanding higher post-exercise expression of miR-486 with protein compared to placebo, there was no effect on NRF-1 expression.

Despite being the most highly expressed of the miRNAs analyzed, there were no post-exercise changes in miR-1 and miR-133a expression. The miR-1 can directly target and inhibit insulin-like growth factor 1 (IGF-I) and Akt-mediated signaling while miR-133 can enhance muscle cell proliferation (Kovanda et al., 2014) indicating putative regulatory roles for these miRNAs in muscle growth/ anabolic responses. Reduced miR-1 expression has been reported following a bout of resistance exercise in human skeletal muscle (Drummond et al., 2008) but aerobic exercise increases miR-1 expression (Nielsen et al., 2010; Russell et al., 2013). It is tempting to speculate that endurance exercise subsequent to resistance exercise counteracted any attenuation of miR-1 expression shown previously following resistance exercise with protein ingestion (Drummond et al., 2008). *In vitro* gain- and loss-of-function studies investigating potential cause-effect between miR-1 expression and IGF-I signal transduction are required to determine any role in the specificity of training adaptation in human skeletal muscle.

In conclusion, this is the first study to characterize miRNA expression following a bout of concurrent exercise in human skeletal muscle and between post-exercise protein and placebo ingestion. We show protein ingestion selectively modulates the expression of several miRNAs with predicted mRNA targets implicated in exercise adaptation responses. Specifically, our findings provide new information on the molecular mechanisms that may mediate enhanced MPS responses following concurrent exercise with protein ingestion. Further research is required to corroborate such a hypothesis and the responses may also be specific to exercise order. Moreover, studies comparing miRNA responses to resistance exercise only and/or protein only interventions incorporating different amounts of protein are needed to further interrogate the effect of concurrent training on miRNAs. Future studies investigating miRNA regulation should also include pathway analysis of all putative targets during/following chronic concurrent training programs to determine the potential for miRNAs to dictate the specificity of training adaptation in skeletal muscle and in maximizing concurrent training adaptation responses.

## Author contributions

DC was involved in conception of study design, completion of exercise trials, experimental analysis, data generation, data interpretation, and preparation of manuscript. JO was involved in data generation, data interpretation, and preparation of manuscript. VC was involved in conception of study design, completion of exercise trials, data interpretation, and preparation of manuscript. JH was involved in conception of study design, data interpretation, and preparation of manuscript.

### Conflict of interest statement

The authors declare that the research was conducted in the absence of any commercial or financial relationships that could be construed as a potential conflict of interest.
